# Standalone RFID Access Control System with Data-Integrity Verification Capabilities

**DOI:** 10.3390/s26092892

**Published:** 2026-05-05

**Authors:** Valentin Popa, Adrian I. Petrariu, Partemie M. Mutescu, Alexandru A. Maftei, Alexandru Lavric

**Affiliations:** 1 Computers, Electronics and Automation Department, Stefan cel Mare University of Suceava, 720229 Suceava, Romania; valentin.popa@usm.ro (V.P.); marian.mutescu@usm.ro (P.M.M.); alexandru.maftei@usm.ro (A.A.M.); lavric@usm.ro (A.L.); 2 MANSiD Research Center, Stefan cel Mare University of Suceava, 720229 Suceava, Romania

**Keywords:** RFID access system, clone detection, data-integrity verification, decentralized architecture

## Abstract

Today, access control systems are used in almost every institution and building. This is because they are an effective solution that provides a high level of security. There are many commercially available systems that provide security-related access features for buildings, including biometric options. Most use a centralized architecture, where each building can be remotely controlled via an Internet connection. This paper presents a completely different system from those on the market, a decentralized system with clone-detection and data-integrity verification mechanisms that allows access to buildings. The overall architecture includes hardware encoding of the access system’s location, and access is granted based on information written to the RFID card by the card-issuing center. This allows the system to be easily reconfigured at the hardware level prior to installation in the access area. The proposed system uses a confidential RFID card data integrity algorithm that uses the card data and immutable UID to determine a checksum in order to validate the RFID card data. As a result, any unwanted modification of at least one bit invalidates the card and blocks access to the building. The system was implemented, validated, and extensively tested over a one-year period with no reported operational issues.

## 1. Introduction

Access control systems are essential components that are part of the security infrastructure in modern buildings. They are commonly used in various settings, including schools, residential complexes, university campuses, hospitals, and industrial sites [1]. An access control system includes technologies and procedures that manage and monitor who can enter or leave a building, ensuring that only authorized individuals gain access. This can be achieved through different methods such as Discretionary Access Control (DAC), Mandatory Access Control (MAC), Role-Based Access Control (RBAC), and Rule-Based Access Control (RuBAC) [2]. These systems operate based on specific rules set by an administrator that determine the access policies.

One of the most common access control methods is radio frequency identification (RFID). RFID is a wireless communication technology that enables an RFID reader to communicate with a specific RFID card [3]. RFID-based access control systems can vary in complexity depending on the application, implementation method, and security requirements. Often, these RFID systems involve connecting to a database queried for each access request from multiple distributed RFID readers. However, using a directly connected database creates a centralized system architecture, which can result in single points of failure. If problems such as database cyberattacks occur, the entire system could become nonfunctional and block RFID card access to the building [4]. Some commercial systems use a local database at the RFID reader level, enabling new users to be added or removed with system-specific RFID cards. However, for this type of system, using them for large deployments would require manually programming each RFID reader with the user list, which is impractical for extensive access systems.

Therefore, to avoid issues associated with centralizing the RFID-based access control system by connecting all RFID readers to a single central unit, and to address the scalability limits of RFID reader-level databases, this paper presents a standalone RFID access solution that offers the advantages of a centralized RFID system while keeping each RFID device independent. In this design, the RFID card contains the authorization data. Access permissions are stored directly on the card, allowing each reader to verify access locally without depending on a central database or network. When the card is presented, the reader retrieves the permission data and determines if the user is authorized. This approach removes the need for continuous server communication and improves scalability. By embedding authorization data in the card, the system can support many access points with minimal infrastructure. Local verification also ensures the system remains functional during network outages or communication failures. Furthermore, if an access control system for a building malfunctions, this does not affect the functionality of the other systems in any way.

The main contributions of this paper are as follows:

- We propose a distributed RFID Control Access System that has the benefits of a centralized system while maintaining each RFID unit independently;
- We propose a novel data allocation scheme for RFID card usage in access control systems, making the system scalable, with clone-detection capabilities and data-integrity verification;
- We implement and extensively evaluate the proposed system in a real-world access management application in a university campus scenario for one year.

The rest of this paper is organized as follows. Section 2 discusses the main challenges related to current RFID access control systems. Section 3 describes the overall system architecture and its components, covering the design from the physical layer through the higher processing pipeline to the application layers. Section 4 details the hardware implementation of the proposed system in a ready-to-use setup and explains its deployment in a case study of a University Campus access system. Finally, Section 5 summarizes the paper’s conclusions and explores potential future developments for the proposed system.

## 2. Challenges on RFID Control Access Systems

Several RFID-based access control systems have been proposed in the literature. In this section, we highlight solutions that combine RFID technology with complementary systems to provide effective access control in a wide range of use cases.

Noprianto et al. [5] proposed an RFID-based access control system with a centralized design where users must register their RFID cards to gain access. The RFID card is scanned at each entry point with an RFID reader, which transmits the data to a database to identify the user and either grant or deny access. Araújo et al. [6] proposed an RFID-based access system in which data such as time, date of access, and the RFID tag ID are sent to an online database for storage. Access control is managed through an Administrator card, which must be presented to the RFID reader to enable user and access management. Salfian et al. [7] designed and implemented a smart access control system that allows remote, real-time access through the integration of IoT and RFID technologies. The access list is stored locally and can be transmitted to a Telegram application via an API to provide various notifications. Al-Imran et al. [8] proposed an RFID-based smart access control system integrated with IoT technology. In the proposed system, the RFID card serves as a credential identification tool, employing dual encryption with the Vigenère algorithm [9] and Bcrypt [10]. Leyu et al. [11] proposed an access control system that uses biometric data. The system employs the open-source OpenCV and Dlib libraries to capture facial features. Additionally, hardware modules like the AS608 [12] are used to capture and process user fingerprints. Biometric data is stored on RFID cards, which are read and interpreted by an RFID module, completing the authentication process within the access control system. A similar approach is presented by Beqqal et al. [13], who propose a multimodal authentication system that integrates RFID technology with biometric methods, including fingerprint and facial recognition. RFID cards enable quick identification by linking each card to a unique ID. Fingerprint verification provides biometric data, while facial recognition enhances reliability across different angles and lighting conditions. Identity validation uses a threshold-based process to balance false acceptance and rejection rates (FAR and FRR). A similar method was presented by Haixia et al. [14].

Suethanuwong et al. [15] proposed an access control system that combines RFID and facial verification technology. In this system, users enroll through a registration process that requires a university account and an RFID card. When access is requested, the user scans their RFID card, followed by facial verification. If both verification steps are successful, access is granted. Additionally, the architecture integrates with the university system through a web service, enabling data retrieval and administrative monitoring of access activities. Suleiman et al. [16] proposed a risk-adaptive RFID access control system based on a multilevel fuzzy inference system. Their solution extends traditional RFID-based approaches by incorporating fuzzy logic [17] to assess risk during access decisions. The system features multiple inference levels, such as authorization, anomaly detection, and risk assessment, analyzing user behavior and access patterns to identify abnormal activity. Niu et al. [18] proposed an access control scheme that combines multi-factor authentication with attribute-based access control. It uses a two-factor authentication protocol integrating RFID technology and facial recognition to ensure high security. To improve authentication efficiency and RFID data processing, elliptic curve cryptography (ECC) [19] is employed. Additionally, a policy index structure is introduced to enhance conflict detection and simplify policy searches, achieving both strong security and high performance.

The systems discussed in this section combine RFID technology with other mechanisms, including biometric authentication, IoT connectivity, fuzzy inference, and centralized database management. While these methods add functionality and security, they also increase hardware needs, software dependencies, and operational complexity. A common issue with these solutions is their reliance on centralized, connected infrastructure for verifying identities and managing data. This dependence creates potential single points of failure, risking system outages due to server failures, network disruptions, or cyberattacks. Additionally, the need for continuous connectivity can limit their use in environments with unreliable or unavailable network access.

In contrast, the system proposed in this paper employs a fully offline and decentralized architecture. Access decisions are made locally, directly at the entry point, without depending on external database connections, cloud services, or internet access. This reduces the risks linked to centralized systems and boosts operational resilience. Additionally, the proposed solution avoids complex or computationally demanding mechanisms, resulting in a lightweight and efficient design. This simplicity lowers implementation costs, enhances reliability, and reduces maintenance needs, all while providing data-integrity verification and clone-detection in an efficient access control system. Table 1 provides a comparison of the literature implementations with the proposed system.

## 3. System Architecture

Most commercial RFID access systems for large-scale deployments, such as campuses or enterprises, use a centralized architecture. All access control units are linked and managed by a main system, usually over a dedicated Ethernet network. This separate network is maintained for security reasons, requiring extra cabling and resources. Access decisions rely solely on the RFID card’s unique identifier (UID), with permissions managed through the central database.

The system presented in this paper features a decentralized architecture that separates individual access control units while enabling users to open all building doors within the deployed access system using a single RFID card. Our system consists four components: an access control unit (ACU), a card writer unit (CWU), a radio-controlled doorbell unit (RCDB), and a graphical user interface (GUI) for managing the RFID card issuance process.

In this architecture, the RFID card has the role of an access token rather than a secure storage element, and therefore confidentiality of card memory is not assumed. The threat model considers attackers capable of reading RFID card memory and UID values using common RFID devices and attempting to duplicate RFID card data. Therefore, the system is designed to detect two types of attacks. The first type of attack is the one in which the card data is modified, the detection being performed by checking the checksum. The second attack is the use of cloneable cards with modifiable UID, which are detected by the ACU by testing the rewriting of the UID. Scenarios such as compromise of the CWU, misuse of the GUI or database by authorized personnel, theft or sharing of valid RFID cards, and advanced card emulation attacks beyond UID-mutable clone cards, are not addressed in this paper. These aspects are considered outside the scope of the proposed system.

The system uses standard ISO/IEC 14,443 [20] Type-A cards with hardware-coded UID values and a trusted ACU executing the verification logic. To protect stored access data, the system employs a deterministic checksum-based integrity mechanism that binds the encoded permissions to hardware coded RFID card parameters. During authentication, the ACU verifies this checksum, and any modification of the stored data results in rejection of the card. This mechanism detects unauthorized data modification and straightforward memory cloning attempts on standard cards.

**Figure 1:**
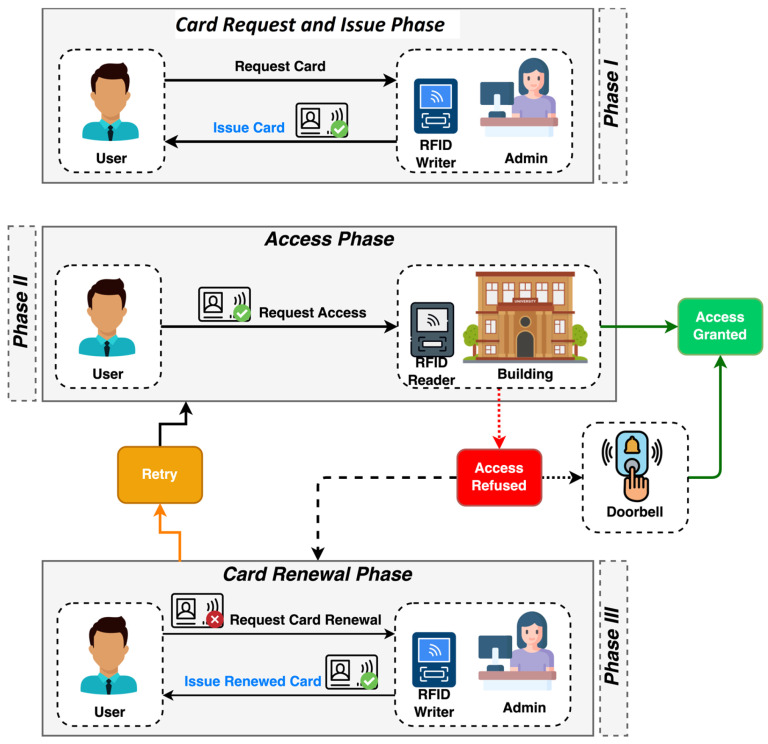
Proposed RFID access system architecture.

As shown in Figure 1, our system’s architecture comprises three main phases: (I) card request and issuance, (II) access, and (III) renewal. The architecture’s entities and components include users, the administrator (which encompasses the CWU and GUI), the RFID card, the ACU, and the RCDB. The initial step in the system involves the administrator giving a card to the user. The administrator provides an RFID card that specifies which buildings and doors the user can access. This RFID card is written and read by a specialized CWU, which is available only to the administration. Once the access list is configured, the card is handed to the user with a limited validity period that can be adjusted through the GUI.

The second stage is known as the access phase. In general, using the RFID card issued by the management entity, the user simply needs to bring the card close to the ACU, and the system will either approve or deny access to the building based on the card’s data. If the user is authorized, no additional input is necessary. If access is denied based on the RFID card information, the user can use the RCDB to request access from a third party inside the building. The doorbell feature is built into the ACU and connects to the RCDB module inside it. The ACU must perform a series of specific card verification steps to determine whether it meets all access requirements. First, the ACU checks whether the card is cloneable or if the UID is hardcoded by attempting to rewrite the UID on the RFID card itself. If the UID can be rewritten by the ACU, the card is automatically rejected without further verification. Next, the reader confirms the card’s validity by comparing the local date from a Real-Time Clock (RTC) module with the card’s valid period. In the final step, it verifies whether the card grants access to the building by querying the blocks that contain the building’s access list.

In the third phase, the user must update their access list on the card. This phase occurs when the user is denied entry to one or more buildings due to expired card validity or lack of access to a specific building. During this phase, the administrator decides whether to renew the user’s RFID card or grant access to a particular building.

A. Access Control Unit

**Figure 2:**
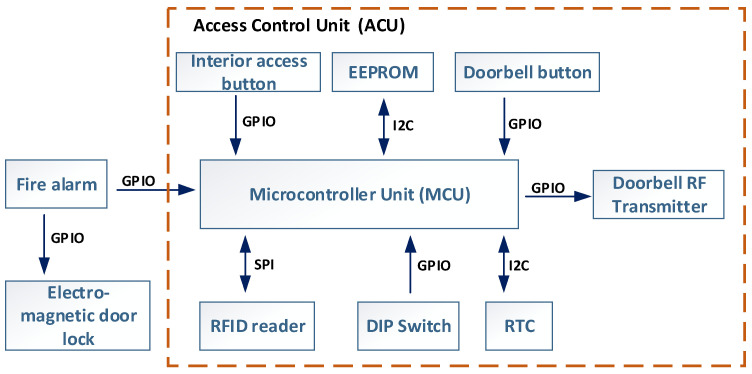
Access Control Unit block diagram.

Figure 2 shows the design and functions of the ACU. The main component of the ACU is a microcontroller (MCU) that manages and coordinates all parts. It uses dedicated GPIOs and data buses, including SPI and I^2^C, to connect to peripheral devices and handle data processing tasks.

The ACU features several peripheral components that support its functions. The main peripheral is the RFID card reader module, which operates at 13.56 MHz using the ISO/IEC 14,443 Type-A communication standard and connects to the MCU via SPI. The RFID antenna is integrated into the ACU’s PCB and calibrated for a 5 cm reading range. An external RTC, connected via I^2^C, handles timekeeping and supplies date and time data to verify RFID card expiration. The RTC uses a 220 mF supercapacitor backup to maintain operation during power outages. The ACU also includes an EEPROM to log fault states and time-stamped data for maintenance and debugging.

Each ACU is identified by its deployment location. This information is programmed into the device using dual-in-line package (DIP) switches, allowing a single firmware to be used across all units. The positions of these DIP switches determine the building and door setup, meaning the location where the ACU is deployed, making it easy to configure the ACU for its specific site. These settings are managed through software that interacts with the hardware configuration. This method also allows units to be reprogrammed and redeployed as needed without changing the embedded software from the MCU, providing flexibility for use in different areas. In the current ACU version, the DIP switches can configure up to 32 buildings (5 bits) and up to 7 individual doors per building (3 bits). These can be extended by adding additional DIP switches, as each switch doubles the number of configurable buildings or doors. An advantage of this design is that the ACU does not rely on a centralized database for access permissions. Instead, it enforces restrictions locally through firmware and hardware settings based on its location.

**Figure 3:**
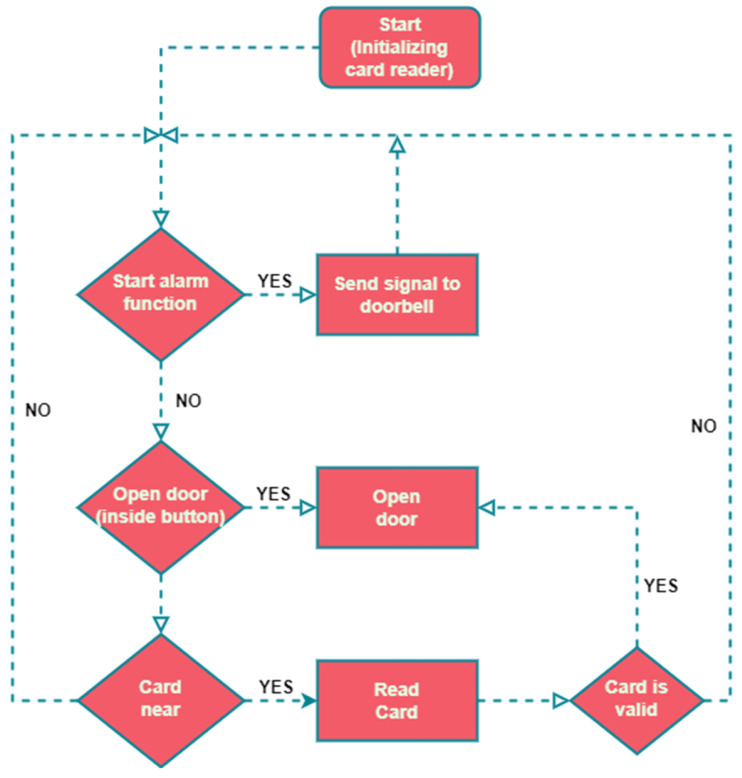
Access Control Unit processing workflow.

The ACU features a doorbell button connected to the MCU via a GPIO pin. When pressed, it sends a request to the paired RCDB using a 433 MHz radio module. To improve receiver selectivity and system security, the data packet includes a unique sequence number, ensuring that only the designated RCDB responds. The ACU can control either an electrically operated mechanical lock or a magnetic lock, depending on the application. In emergencies, a Fire Alarm input automatically unlocks the door, ensuring the system does not hinder evacuation. To enhance reliability, the ACU features a power backup module that can operate for up to 12 h during power outages. The software logical diagram for this component is based on the block diagram shown in Figure 3.

During the initialization phase, the ACU reads the building and door parameters based on the DIP switch configuration. Then it checks the EEPROM for any recorded fault states and verifies whether the RTC module is synchronized. Once these initial checks are complete, the ACU begins its operational cycle. The loop starts by checking the doorbell button status. If pressed, it sends a command to the paired RCDB via the radio module and plays a confirmation sound to alert the user that the doorbell command has been sent. If not pressed, the ACU then checks the inside door lock button. If no door-opening command is received from the inside door button, the ACU checks for an RFID access card. If no RFID card is detected, the program loops back to the start. When a card is detected, its contents are read and its validity is verified. If access is granted, the ACU deactivates the door lock mechanism for 4 s. If access is denied, the door remains locked. In either case, the ACU provides feedback to the user through audible signals (using an internal buzzer) and visual cues (with status LEDs) indicating the result of the access attempt.

B. Radio-controlled Doorbell

The RCDB module is designed for use at security guard observation posts near main building entrances where controlled access is necessary. This module receives radio signals from the ACU when the doorbell button at the entrance is pressed. When it receives the signal, the RCDB plays a notification sound, prompting the guard to assist at the access door. The RCDB is particularly useful in scenarios when:

- a user with access has forgotten their RFID card inside the building;
- a visitor without access requests entry to see someone in the building.

**Figure 4:**
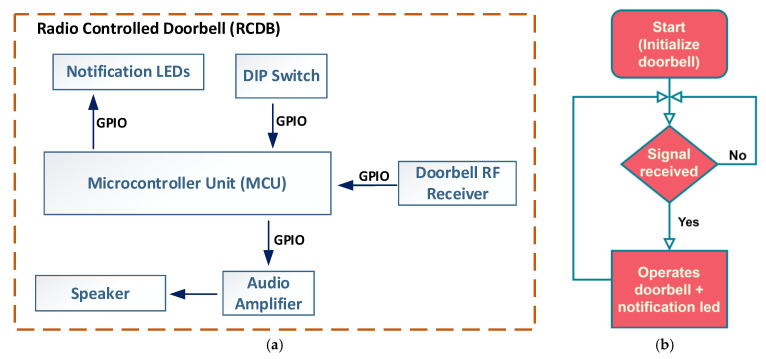
(**a**) Radio-controlled doorbell block diagram; (**b**) Radio-controlled doorbell processing workflow.

If a doorbell ring command is sent from the ACU to the RCDB, the module will activate a sound alert and display a light notification. The light stays on until the door is unlocked, either with a valid RFID card or by pressing the inside access button. This feature ensures continuous visual notification when the guard is not immediately present upon the doorbell ringing, helping to maintain awareness of pending access requests. The block diagram of the RCDB hardware components is shown in Figure 4a.

The primary component of the RCDB is a microcontroller unit (MCU) that manages communication with all peripheral devices. The radio receiver module connects to the MCU via a GPIO pin. When a doorbell command is received, the MCU checks the data packet to see if it is addressed to the paired RCDB or another unit. If the packet is for the RCDB, the MCU activates an audible alert, which is amplified and played through the speaker. Additionally, the MCU controls the status LEDs to show the device’s current state, providing visual feedback to users or security personnel. Figure 4b illustrates the RCDB processing workflow. During setup, the DIP switch configuration allows the RCDB to connect with its designated ACU and creates an identification sequence to verify incoming radio data packets. This method also enables multiple ACUs to pair with a single RCDB, multiple RCDBs to pair with a single ACU, or multiple RCDBs with multiple ACUs.

C. RFID Access Card

**Figure 5:**
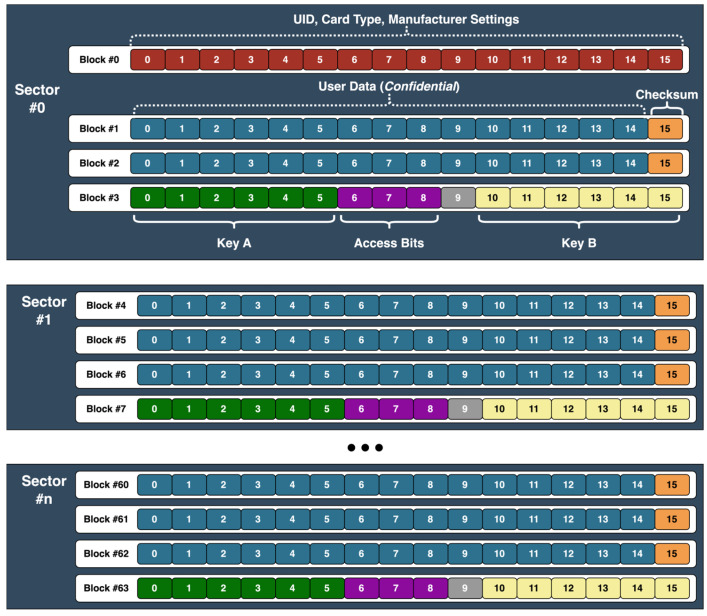
Proposed RFID data card organization.

The RFID access card used in the proposed campus access system operates at 13.56 MHz and complies with the ISO/IEC 14,443 Type-A standard. The current implementation uses MIFARE Classic cards, which internally implement the Crypto-1 [21] protocol. However, the proposed system does not rely on the cryptographic strength of this protocol, as card data integrity is verified at the application level by the Access Control Unit. The card has a total memory capacity of 1 kB, which is used to store RFID card data, user information, and access list details. The clone-detection and data-integrity verification features are not inherent properties of the RFID card itself but are part of the proposed data allocation scheme. A graphical representation of the organization of the RFID Access Card data is shown in Figure 5.

The RFID card’s memory structure has 16 sectors, each with four 16-byte data blocks. In the first sector, block zero holds manufacturer information, which is fixed on non-cloneable cards, while another contains security access keys. All other sectors reserve one block for security keys, leaving 47 data blocks (752 bytes) available for custom data storage. In our system design, two data blocks are allocated to store user information and the card’s expiration date. The last byte of each remaining data block is reserved for a checksum calculated using a confidential proprietary algorithm based on the card’s stored data. Because the checksum depends on all stored data, even a single-bit modification anywhere on the RFID card results in a checksum mismatch, flagging the card as invalid. This mechanism enables data-integrity verification and detects unauthorized modification without knowledge of the checksum formula.

Following these allocations, 675 bytes are still available for storing door access permissions. Each bit represents access to a specific door (0 = no access, 1 = access granted), allowing up to 5400 permissions to be stored on a single RFID card. The checksum system ensures the integrity of the encoded access data and prevents unauthorized modifications. To formally describe the integrity verification mechanism, let D denote the encoded access data stored on one of the RFID card blocks, and C the associated checksum value. The checksum is determined using a deterministic function of the stored data and hardware coded RFID card parameters, such as UID. The checksum function can be abstractly represented as C=f(D,UID). During authentication, the ACU reads D and C, recomputes $C^{’}=f(D,UID)$, and verifies that $C^{’}=C$. If the values do not match, the card is automatically rejected.

The checksum function has three clear properties. First, it is deterministic, meaning that for the same D and the same UID of the card, it always generates the same value. It is tied to the UID of the card, so the same data cannot be copied to another card without losing its validity. Also, all the data is used for calculating the checksum, so any change, even of a single bit, leads to inconsistencies.

We can assume that an attacker can read the entire contents of the card memory and the UID. However, the checksum formula is not stored on the card and is known only to the ACU and the CWU. This means that the attacker can copy or modify the data but cannot calculate a valid checksum for the modified data. When the ACU recalculates the checksum, the values will not match, and the card will be rejected.

From a security perspective, this encoding scheme provides two main advantages: (I) high storage efficiency through dense bit packing, where doors and buildings are represented as individual bits, and (II) integrity protection via checksum validation. These features help to ensure that access policies remain consistent, verifiable, and able to detect unauthorized data modification, even without centralized validation. In addition to safeguarding data integrity, clone detection mechanisms are implemented at the ACU level. When a card is presented, the ACU tries to overwrite the RFID card’s UID field. Genuine cards have factory-set, unchangeable UIDs, while cloneable cards permit UID modifications. If the overwrite operation succeeds, the card is identified as cloneable and is immediately rejected without further processing. This validation provides an additional mechanism for clone-detection, supplementing the data integrity safeguards embedded in the card memory structure.

D. Card Writer Unit

The CWU is a tool designed to assist administrators in managing RFID cards. It acts as an interface between the GUI and the RFID card, translating the card’s configuration from the higher application layer into data stored in the card’s physical memory. Besides issuing cards, the CWU reads the current RFID card configuration and compares it with the main local access database, which contains access management details. This process helps to detect data tampering by comparing the local database configuration with the data read from the RFID card, since the database is only updated after a successful write to the RFID card.

**Figure 6:**
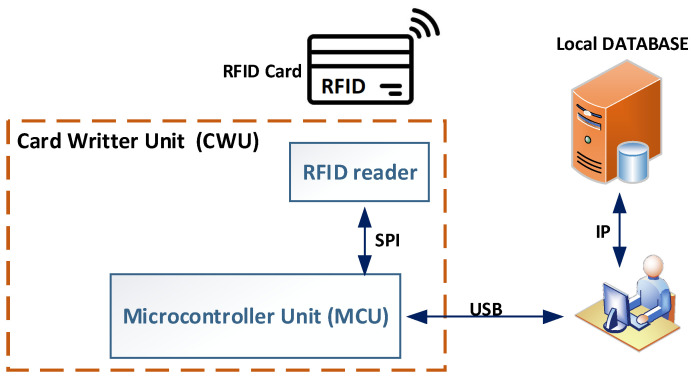
Card Writer Unit block diagram.

The hardware design of the CWU is shown in Figure 6. The unit features an RFID card reader module, like that used in the ACU, connected to the MCU via an SPI bus. The MCU runs firmware specifically developed to interact with the GUI software v1.0, which sends custom commands to enable reading and writing operations on the RFID card. Additionally, the CWU contains the security keys needed to access the data on the RFID card.

**Figure 7:**
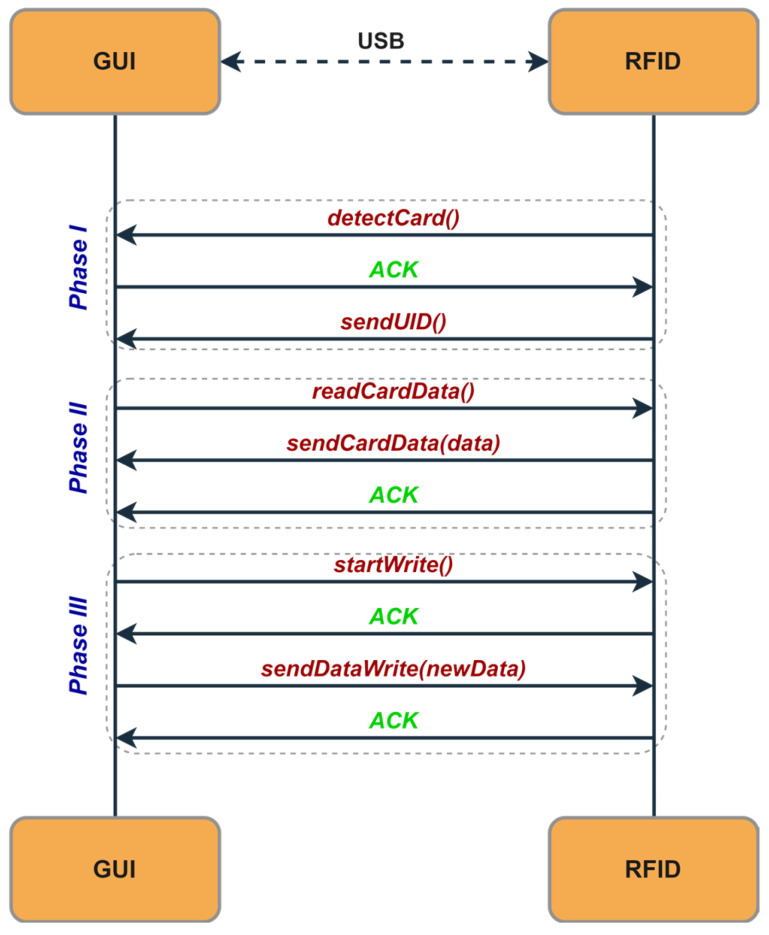
Sequence diagram for GUI–RFID communication protocol.

An example of a sequence diagram between the CWU and the GUI software is shown in Figure 7. When the CWU detects a card, it sends a request to the GUI software, which responds with an acknowledgment (ACK). After receiving the ACK, the CWU transmits the RFID card’s UID to the GUI software for verification against the database. Once the UID is validated, the GUI software can send read or write commands. These operations are synchronized between the CWU and the GUI software using ACK sequences to ensure data integrity.

E. Graphical User Interface (GUI)

The GUI is a flexible software tool that supports various functions within the RFID access system. Its main role is to provide an easy-to-use platform for managing RFID card data. This includes tasks such as granting or revoking access to specific doors, updating the card’s expiration date, and issuing new RFID cards. These tasks are performed through the CWU, which is connected to the GUI to enable data transfer to the RFID cards. When an RFID card is detected, the CWU notifies the GUI application and then waits for an ACK response. After receiving the ACK, it sends the UID of the RFID card to the application and waits for further commands. If a read command is received, the CWU transmits the requested data in blocks, waiting for an ACK after each one. If any errors occur during reading, the CWU sends an error message and then waits for the next command from the application. For the write commands, the CWU confirms receipt by sending an ACK to the application. It then waits for the application to specify the data block index where the information will be written, along with the 16 bytes of data to store. After the write operation, the CWU responds with an ACK if the write succeeds or an ERR if any errors occur.

**Figure 8:**
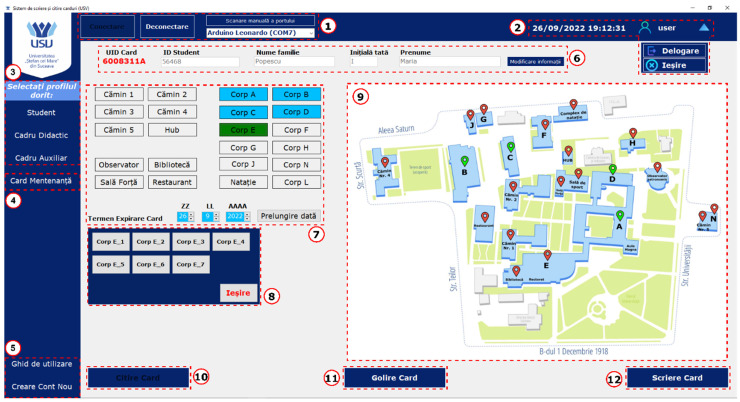
Graphical User Interface for RFID access system.

The GUI functions as an interface between the campus access system and a centralized database. Any action that modifies an RFID card is logged in the database to keep a detailed record of user access. Access to the GUI requires an individual account, and each recorded activity is linked to the specific user who performed it. This ensures clear accountability and traceability for all activities carried out through the system. Figure 8 shows the GUI of the proposed system, developed for a university campus use case, specifically on the Ștefan cel Mare University of Suceava campus. The interface is divided into several functional sections, each designed to support a particular aspect of RFID card management and system operation. To maintain data integrity and prevent incomplete configurations, input validation features are implemented across all sections. Any required fields left blank trigger immediate error messages, ensuring all necessary information is entered before proceeding with card issuance or modification.

The interface adjusts based on the recipient’s role, whether Student, Teacher, or Auxiliary Staff.

- **Section 1:** This section includes controls for connecting or disconnecting the GUI from the CWU. While the application allows manual connection to the RFID card reader and writer, it is mainly designed to detect, display, and automatically connect to the device.
- **Section 2:** This area offers user account controls and shows the current date and time. These features ensure actions are logged and linked to the correct user, maintaining accountability for access with a specific card.
- **Section 3:** Users can choose a specific profile to preload a list of access permissions, with three available modes: Student, Teacher, or Auxiliary. Only one profile can be assigned to each card.
- **Section 4:** This part discusses creating a special maintenance RFID card that enables solving ACU fault states with minimal manual effort and without needing direct access to the system’s internal hardware.
- **Section 5:** This section offers a user guide on navigating and using the GUI. It also includes tools for creating new user accounts, making sure only authorized personnel with an account can add new accounts.
- **Section 6:** Users can enter the cardholder’s information, such as student ID, first name, last name, and the UID of the RFID card currently on the CWU. These details are necessary to link the card to its owner within the local database.
- **Section 7:** This section displays a list of campus buildings where access can be granted or revoked. Buildings that the cardholder has access to are highlighted in a distinctive color. This section also includes options to set the card’s validity date, which defaults to one academic year but can be extended for special cases.
- **Section 8:** This section extends Section 7 by listing all doors in each building, allowing for control over access permissions.
- **Section 9:** This part features a visual map of the campus. Buildings accessible to the cardholder are clearly marked for easy identification.
- **Section 10:** This section features the Read Card button. While the RFID card is automatically read when placed on the CWU, users can also manually initiate a RFID card reading process using this button.
- **Section 11:** This section permits users to erase an RFID card entirely, removing all stored data.
- **Section 12:** The final section includes the “Write Card” button, which sends the configured access data to the RFID card, completing the card-writing process. After the writing is finished, an automatic read process is initiated to verify if the data on the card matches the stored configuration in the local database.

Each element of the graphical user interface is designed to make RFID card management easier. The application includes security features, such as a dual-verification system for RFID cards. This provides an extra layer of security for GUI users, ensuring precise operations and minimizing human errors.

F. Administrative Model and Credential Management

The CWU and GUI are deployed in a trusted administrative environment and are accessible only to authorized personnel responsible for issuing and managing RFID credentials. The CWU stores the security keys required to access the RFID card sectors and performs card writing operations, while the ACUs are limited to reading and validating card data. This way, RFID card generation and modification are restricted to the administrative layer.

Card revocation and lost-card handling are performed through the GUI and the associated database that maintains the mapping between card UID and user identity. When a card is reported lost, the corresponding record can be invalidated and a replacement card issued. The GUI records card issuance and modification events to provide traceability. More advanced features, such as audit logging and automated revocation propagation, are not yet implemented and are considered future work.

## 4. Hardware Implementation

**Figure 9:**
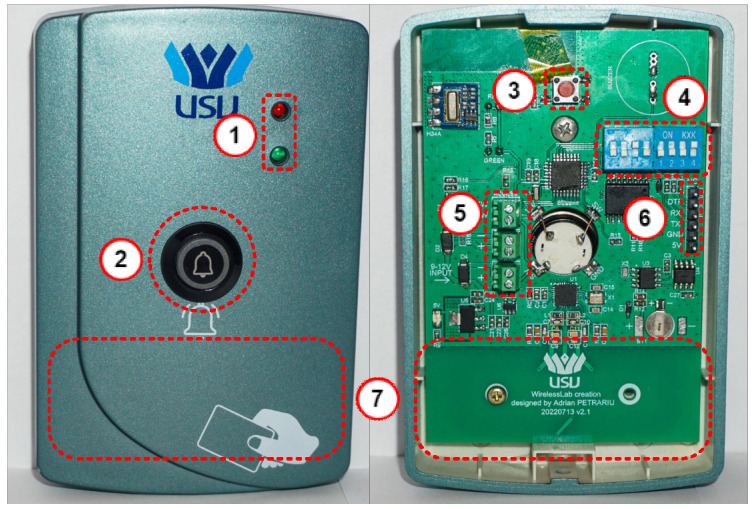
Access control unit hardware implementation.

This section describes the hardware implementation of the proposed RFID Access Control System. Figure 9 illustrates the hardware setup of the ACU, which includes the following components: LEDs for status indication (1); doorbell button (2); system reset button (3); building/door hardware programming switches (DIP switches) (4); connectors for power supply and entrance access button (5); UART port for microcontroller programming, firmware upload, and system debugging (6); and RFID antenna area (7).

The ACU’s operating modes are indicated by its status LEDs. In standby mode, the red LED remains on, showing the unit is active and ready. When a valid RFID card is scanned, the red LED turns off, and the green LED lights for 4 s, confirming successful authentication. Afterwards, the device automatically returns to standby. If an invalid card is detected, the red LED blinks three times, with each blink accompanied by a sound to alert the user of the rejection.

The ACU has two fault modes, which are indicated by the status LEDs. The first fault mode happens when the internal RTC becomes out of sync. This results in a notification rather than a critical error, so the device still functions normally for card reading, button use, and doorbell functions. To fix this, the RTC can be synchronized using a maintenance RFID card from the RFID card issuance center after specifying the building where the ACU is installed. Successful resynchronization is shown by both status LEDs blinking three times, each with an audible beep. This resync process can also be started manually by holding the doorbell button and bringing the maintenance card close to the reader. The RTC module was chosen to meet the system’s outdoor mounting needs, using the DS1307ZN+ [22], which operates from −40 °C to +85 °C. The backup supercapacitor for the RTC is the DSK-614, which keeps the RTC active during a system power failure. It has a capacity of 0.2 F, a maximum voltage of 3.3 V, and an equivalent series resistance (ESR) of 4 kΩ or less, according to the datasheet [23], and can support the system RTC for more than 10 days. This backup supercapacitor can also operate normally between −10 °C and +60 °C with all parameters within the normal range. The backup supercapacitor is powered from the main system power supply through a Schottky diode with a leakage current of only 2 nA.

**Figure 10:**
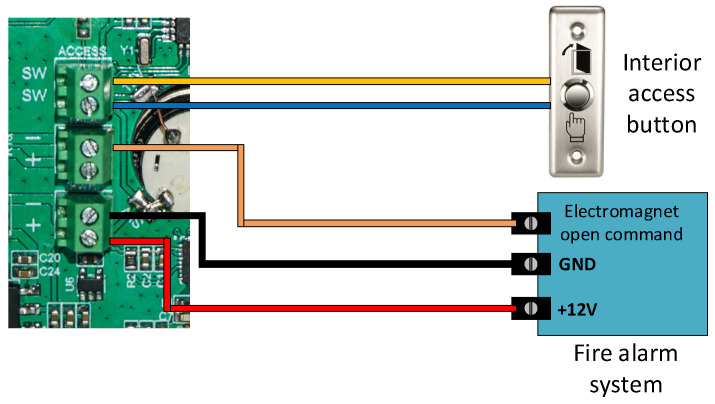
Access control unit door lock control interfaces.

The second fault mode indicates that the ACU’s DIP switches have not been configured for a specific building or door. In this state, the red LED remains steadily on, while the green LED flashes once every two seconds, with each flash accompanied by a beep. To resolve this problem, the DIP switches must be correctly configured, and the device should be reset. The DIP switches are controlled through an 8-bit I/O expander. The ACU can be installed with its own door lock or integrated into the building’s lock system to send locking and unlocking commands. Figure 10 shows how the ACU connects to the building’s centralized system, which also includes the fire alarm system. This configuration allows the ACU to operate in different setups to suit the building’s needs.

**Figure 11:**
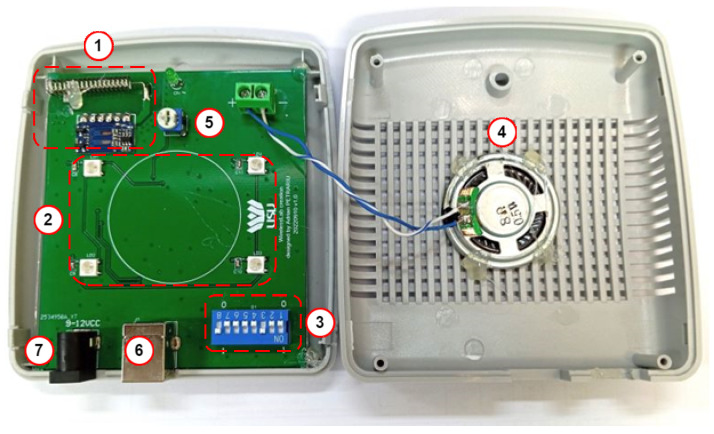
RCDB hardware implementation.

Figure 11 shows the hardware layout of the RCDB module. The PCB features a conveniently accessible potentiometer for volume adjustment (5) of the speaker (4) and four RGB LEDs (2) that indicate access to the doorbell button from the ACU. The LEDs stays on and blink at 2 s intervals until the magnetic lock engages and the door opens. The radio module’s (1) RX antenna is horizontally polarized to align with the ACU’s TX antenna. Pairing the device with the ACU involves setting the DIP switches (3) identically on both devices, allowing RCDBs to be paired with different ACUs without reprogramming. The RCDB is powered through a USB Type-B connector (6), chosen for its durability and reliability over time, or via a standard 2.1 mm power supply connector (7). Additionally, the same USB Type-B connector is used for programming the RCDB.

**Figure 12:**
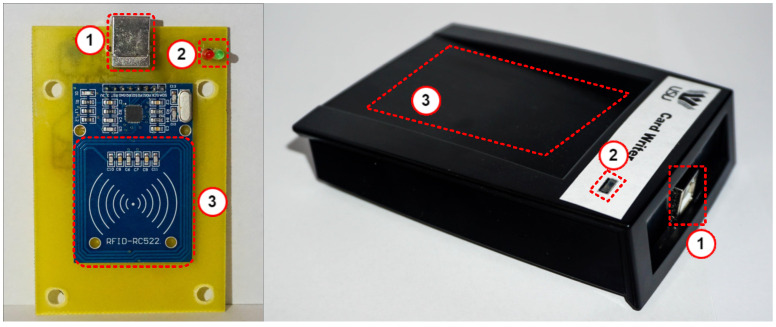
Card writer hardware design.

Figure 12 shows the hardware layout of the CWU. It includes a USB Type-B port for connecting to a PC and providing power (1), and status LEDs to display RX and TX activity (2). The RFID antenna area (3) is also included. The card writer works with a GUI to manage user access and configure RFID cards. After laboratory validation, the standalone RFID access control system was deployed across several buildings on the Ștefan cel Mare University of Suceava campus. The system was tested for roughly 12 months under actual conditions, which included various environmental factors and high daily usage. During this period, no critical system failures were reported, and all ACUs remained stable without needing hardware interventions.

**Figure 13:**
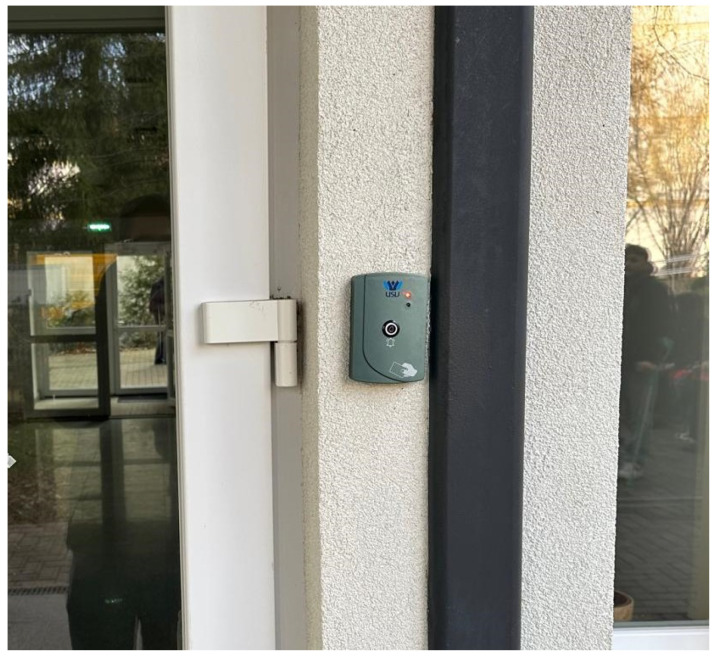
ACU installed at the entrance of a university dormitory building.

The decentralized architecture allowed continuous operation during network outages, illustrating the system’s reliability in real-world use. Figure 13 shows an ACU installed at the entrance of a campus dormitory housing approximately 300 students. Based on typical daily activity, with each resident making three to four access attempts, the system is expected to handle between 900 and 1200 authentication requests each day at this entrance. This operational demand further highlights the system’s capacity to support and control building access without any drop in performance.

To quantitatively evaluate system performance, a series of controlled experiments was conducted over 10,000 authentication attempts. The authentication latency, measured through the UART debugging interface, had an average value of 121 ms, with a maximum observed latency of 187 ms. The card read failure rate was below 1%, with most failures caused by improper card positioning on the RFID reader. Security validation tests showed a 100% rejection rate for unauthorized cards, including tampered cards with modified data and expired credentials. To evaluate the rejection of UID-cloneable cards, an additional set of 200 authentication trials was performed using MIFARE Magic RFID cards with changeable UIDs, onto which legitimate RFID card contents had been fully cloned. This evaluation also resulted in a 100% rejection rate, since the MIFARE Magic cards responded to the UID modification request of the ACU and were immediately identified as cloneable and rejected without further processing. The distribution of test scenarios is summarized in Table 2.

Additionally, a power outage scenario was tested, where the ACU was disconnected from power for a duration of 8 h; which, according to [24], is the average power outage time during major events. The backup capability of the ACU was evaluated using the integrated 0.2 F supercapacitor. The RTC has a backup current requirement of 500 nA and a usable voltage range between 3.3 V and 1.25 V, the supercapacitor can theoretically sustain RTC operation for approximately 9.5 days. This duration significantly exceeds the tested outage period. During the experiment, no loss of timekeeping data or desynchronization was observed after power restoration, with the ACU boot-up time of approximately 2 s.

## 5. Conclusions

This paper presents a distributed RFID-based access control system that overcomes key limitations of traditional centralized solutions in large-scale environments like university campuses. Unlike conventional systems that depend on a central database or constant network connection, the proposed system stores authorization data on the RFID card and verifies access locally at each Access Control Unit (ACU). This design preserves the scalability and usability of centralized access control while allowing each access point to operate independently.

The system consists of four main parts: the ACU, Card Writer Unit (CWU), Radio-Controlled Doorbell (RCDB), and administration GUI, which work together as a complete access management system for issuing, validating, updating, and managing RFID credentials. A key feature is the RFID data organization scheme, which enables compact encoding of access permissions and includes data-integrity verification and clone-detection capabilities. By using bit-level permission storage, checksum-based integrity checks, and UID mutability verification at the ACU, the system can detect unauthorized data modifications and reject UID-cloneable cards.

The hardware implementation demonstrates the practicality of the proposed architecture. The system operated continuously for about 12 months (over 900 daily accesses granted per day) at the Ștefan cel Mare University of Suceava campus without any critical failures. This deployment demonstrated that the decentralized design supports high-demand authentication scenarios, remains active and reliable during communication outages, and requires minimal maintenance. These results confirm the solution’s feasibility for real-world campus access control.

Another contribution of our work is the design of a novel data allocation scheme for the use of RFID cards in access control systems, which makes the system scalable, with data-integrity verification and clone-detection capabilities.

Future work will focus on enhancing the system’s capabilities. Key areas include adding telemetry to the ACUs using secure, end-to-end encrypted wireless technology such as LoRaWAN, enabling faster revocation of lost or stolen cards, expanding logging and auditing capabilities, and connecting to larger smart campus ecosystems.

## Figures and Tables

**Table 1:** Comparison of RFID-Based Access Control Systems.

System	Authentication Mechanism	Infrastructure Dependence	Cost	Scalability	Revocation	Maintenance	Offline Operation
Noprianto et al. [5]	RFID UID + central DB	High (database server)	Medium	Moderate	Centralized	Server maintenance	No
Araújo et al. [6]	RFID + online logging	High (online database)	Medium	Moderate	Centralized	Database and network	No
Salfian et al. [7]	RFID + IoT notifications	High (cloud/API)	Medium	High	Centralized	IoT infrastructure	Limited
Al-Imran et al. [8]	RFID + encryption	Medium–High	Medium	Moderate	Centralized	Software maintenance	Limited
Leyu et al. [11] Beqqal et al. [13]	RFID + biometrics	High (multiple sensors)	High	Moderate	Centralized	Sensor calibration	No
Suleiman et al. [16]	RFID + fuzzy logic risk model	High	High	Moderate	Centralized	Model tuning	No
Niu et al. [18]	RFID + ECC + MFA	High	High	Moderate	Centralized	Cryptographic infrastructure	Limited
Proposed system	RFID + checksum integrity	Low (local ACU)	Low	High	Local revocation	Low	Yes

**Table 2:** Summary of experimental authentication scenarios and observed results.

Test Category	Number of Samples	Observed Result (Successfully Read RFID Cards >99%)
Valid Cards	7000	100% accepted
Tampered data cards	2000	100% rejected
Expired Cards	1000	100% rejected
UID-mutable clone cards	200	100% rejected

## Data Availability

No new data were created or analyzed in this study. Data sharing is not applicable to this article.

## Abbreviations

The following abbreviations are used in this manuscript:

RFID	Radio Frequency Identification
API	Application Programming Interface
UID	Unique Identifier
ACU	Access Control Unit
CWU	Card Writer Unit
RCDB	Radio-Controlled Doorbell
GUI	Graphical User Interface
RTC	Real-Time Clock
MCU	Microcontroller Unit
SPI	Serial Peripheral Interface
ISO	International Organization for Standardization
IEC	International Electrotechnical Commission
PCB	Printed Circuit Board
EEPROM	Electrically Erasable Programmable Read-Only Memory
DIP	Dual In-line Package
GPIO	General Purpose Input/Output
ACK	Acknowledgment
ERR	Error
UART	Universal Asynchronous Receiver-Transmitter
LED	Light Emitting Diode
ESR	Equivalent Series Resistance
RX	Receive
TX	Transmit
